# Inhibition of Melanogenesis by the Pyridinyl Imidazole Class of Compounds: Possible Involvement of the Wnt/β-Catenin Signaling Pathway

**DOI:** 10.1371/journal.pone.0033021

**Published:** 2012-03-13

**Authors:** Barbara Bellei, Angela Pitisci, Enzo Izzo, Mauro Picardo

**Affiliations:** Laboratory of Cutaneous Physiopathology, San Gallicano Dermatologic Institute, Istituto Di Ricovero e Cura a Carattere Scientifico, Rome, Italy; University of Tennessee, United States of America

## Abstract

While investigating the role of p38 MAPK in regulating melanogenesis, we found that pyridinyl imidazole inhibitors class compounds as well as the analog compound SB202474, which does not inhibit p38 MAPK, suppressed both α-MSH-induced melanogenesis and spontaneous melanin synthesis. In this study, we demonstrated that the inhibitory activity of the pyridinyl imidazoles correlates with inhibition of the canonical Wnt/β-catenin pathway activity. Imidazole-treated cells showed a reduction in the level of Tcf/Lef target genes involved in the β-catenin signaling network, including ubiquitous genes such as Axin2, Lef1, and Wisp1 as well as cell lineage-restricted genes such as microphthalmia-associated transcription factor and dopachrome tautomerase. Although over-expression of the Wnt signaling pathway effector β-catenin slightly restored the melanogenic program, the lack of complete reversion suggested that the imidazoles interfered with β-catenin-dependent transcriptional activity rather than with β-catenin expression. Accordingly, we did not observe any significant change in β-catenin protein expression. The independence of p38 MAPK activity from the repression of Wnt/β-catenin signaling pathway was confirmed by small interfering RNA knockdown of p38 MAPK expression, which by contrast, stimulated β-catenin-driven gene expression. Our data demonstrate that the small molecule pyridinyl imidazoles possess two distinct and opposite mechanisms that modulate β-catenin dependent transcription: a p38 inhibition-dependent effect that stimulates the Wnt pathway by increasing β-catenin protein expression and an off-target mechanism that inhibits the pathway by repressing β-catenin protein functionality. The p38-independent effect seems to be dominant and, at least in B16-F0 cells, results in a strong block of the Wnt/β-catenin signaling pathway.

## Introduction

Melanocytes are specialized cells located at the basal layer of the epidermis that produce and transfer melanin pigments to surrounding keratinocytes, thereby contributing to the appearance of skin color. Within keratinocytes, melanins provide a primary defense system against UV radiation by preventing cellular injury and consequential DNA damage that can cause cancer and aging of the skin [1], [2]. Melanin is produced in specialized organelles named melanosomes that are only observed in pigment cells. In melanosomes, melanins are synthesized via a well-characterized enzymatic cascade that is controlled by tyrosinase, tyrosinase-related protein 1 (TRP1), and dopachrome tautomerase (DCT) also known as tyrosinase related protein 2 (TRP2), and that leads to the conversion of tyrosine into melanin pigments [3], [4]. In particular, tyrosinase plays a key role in this process, because it catalyzed the initial and rate-limiting step of melanogenesis [5]. Melanogenesis is subject to complex regulatory controls by a large number of intrinsic and extrinsic factors that may be produced by the environment or by neighboring cells in the skin. These factors include UV radiation, melanocyte stimulating hormone (MSH) [6], [7], agouti signal protein (ASP), endothelin 1 (ET1), and a wide variety of growth factors and cytokines [8], [9]. The most important transcription factor in the regulation of tyrosinase [10], [11] and tyrosinase-related proteins (TYRPs) [12] is the microphthalmia-associated transcription factor (Mitf). Mitf expression is induced by the activation of the melanocyte differentiation program. In addition, Mitf is a nuclear mediator of Wnt signaling during melanocyte differentiation. The Wnt proteins play multiple roles in the process of neural crest formation, affecting induction, migration, proliferation and differentiation [13]. Mice deficient in Wnt-1 and Wnt-3 lack pigment cells, and this phenotype is probably due to the failure of early neural crest cells to expand properly [14]. In addition to the critical role that β-catenin plays in prenatal melanocyte biology, we recently demonstrated a physical interaction between CREB and β-catenin following PKA/cAMP pathway activation in normal human melanocytes and B16-F0 mouse melanoma cells that led to a functional cooperation of β-catenin and CREB on the *Mitf* promoter [15]. Another hint of the importance of the link between Wnt signaling and Mitf in melanocyte development is provided by evidence showing that β-catenin is not only involved in lymphoid enhancer factor1 (Lef1)-dependent control of *Mitf* gene transcription but also functionally interacts with the Mitf protein [16].

One of the key factors in β-catenin regulation is the control of its stability, which in turn influences its translocation into the nucleus and its binding to T-cell factor (Tcf)/lymphoid enhancer factor (Lef) family transcription factors [17], [18]. Extensive studies have demonstrated that the activity of the β-catenin-Tcf/Lef transcription complex can be regulated by mechanisms independent of Wnt glycoproteins secretion and β-catenin nuclear translocation [19]. Many different nuclear proteins interact with the β-catenin-Tcf/Lef transcriptional complex, resulting in both stimulation and repression of Wnt target genes [20]. The regulation of Wnt signaling by protein-protein interaction is tightly regulated by post-transcriptional modifications such as phosphorylation, ubiquitination and sumoylation [21]. Consequently, the degree to which β-catenin-dependent transcription is regulated is dictated by the availability of β-catenin binding partners, and the phosphorylation of β-catenin can affect some binding interactions with cofactors [22]–[24]. Moreover, the mechanism of switching from differentiated/proliferative to differentiated/invasive melanoma cells phenotype involves the fact that β-catenin engages the two closely related transcriptional factors Lef1 and Tcf4 in a mutually exclusive fashion [25]. Recent studies have proposed the use of both natural and synthetic compounds that modulate β-catenin protein-protein interaction and activity as possible therapeutic options in cancer [26]–[31].

Even if Mitf, that is considered the master regulator of pigmentation, is a target of the Wnt pathway [32], the impact of Wnt/β-catenin pathway modulation on adult melanogenesis and pigmentary disorders has not been studied in detail. In cell culture, specific silencing of β-catenin expression reduces the levels of melanocyte differentiation-associated markers, such as melanin synthesis, tyrosinase activity, and protein expression [15]. High levels of expression of Dickkopf-1 (DKK1), an inhibitor of the canonical Wnt signaling pathway secreted by fibroblasts in the dermis of human skin, is responsible for the low level of pigmentation of the palmoplantar areas of human adults via the suppression of β-catenin and Mitf [33], [34].

Small cell-permeant inhibitors of protean kinases have become precious reagents with which to investigate the physiological roles of protein kinases. ATP-competitive antagonists of the p38 stress-activated protein kinases have been used to assess the physiological role of p38 MAPK in melanogenesis [35], [36]. While investigating the effects of the pyridinyl imidazole compounds SB202190 and SB203580, we found that the inhibitory activity of these compounds on melanin synthesis was independent of p38 MAPK. In this study, in order to characterize the mechanism of melanogenic pathway inhibition, we evaluated the impact of pyridinyl imidazoles on signal transduction pathways mediating melanogenesis. Data presented demonstrate that the inhibitory activity of SB202190, SB203580 and of other structurally-related pyridinyl imidazoles (PI) correlated with inhibition of the canonical Wnt/β-catenin pathway activity and consequent decrease of Mitf expression.

## Results

### Effect of pyridinyl imidazole compounds on melanogenesis

**Figure 1:**
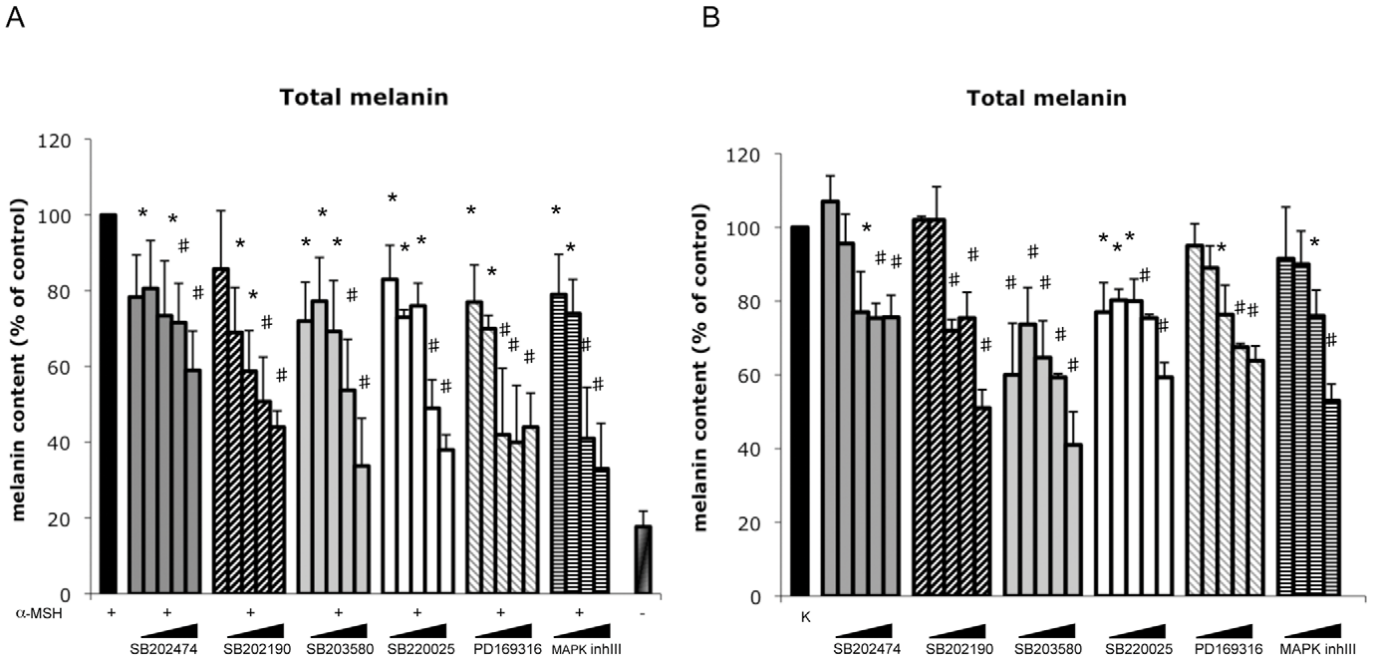
Effect of pyridinyl imidazoles compounds on melanin synthesis in B16-F0 melanoma cells. (A) Following incubation with α-MSH (0.1 µM) and increasing concentrations (1, 2.5, 5, 10, 20 µM) of pyridinyl imidazoles for 72 h, the extracellular and intracellular levels of melanin were determined separately by measuring the absorbance at 405 nm. Standard curves of synthetic melanin were used to extrapolate the absolute values of melanin content. The total amount of melanin was calculated for each experimental point by adding the extracellular and intracellular melanin values after normalization for protein content. Total melanin produced at the end-point by control (DMSO-treated cells) and hormone-stimulated cells (α-MSH plus DMSO-treated cells) is reported for comparison. (B) B16-F0 cells were also treated with pyridinyl imidazoles compounds for 96 h in absence of α-MSH. Results are expressed as percentage of untreated control samples. The data show the mean±SD of three experiments performed in duplicate. *P≤0.05; #P≤0.01 versus control.

Small-molecule inhibitors of p38 protein kinases have contributed greatly to our understanding of MAP kinase biological signaling. However, some evidence showed that pyridinyl imidazole compounds unexpectedly may have unexpected targets in addition to p38 MAPK [37]–[41]. We recently reported that SB202474 (4-Ethyl-2(P-Methoxyphenyl)-5-(4′Pyridyl)-1H-Imidazole), a structural analog of p38 inhibitors SB202190 and SB203580, that is commonly included as a negative control, caused a significant p38-independent inhibition of pigmentation, demonstrating that the imidazoline class of compounds acts through an off-target effect [42]. In this study, in order to characterize the mechanism of melanogenic pathway inhibition, we tested the effect of a selection of pyridinyl imidazole derivatives on both spontaneous and hormonal-stimulated melanogenesis. In presence of α-MSH, a potent and physiological hormonal stimulator of pigmentation, B16-F0 murine melanoma cells were analyzed after 72 h of co-treatment, whereas unstimulated cells were treated with compounds for 96 h before analysis because spontaneous melanogenesis is a much longer process. We tested SB202474, SB202190, SB203580, SB220025, PD169316 and p38 MAPKinase inhibitor III on α-MSH-treated cells and assayed their dose-dependent (1–20 µM) reduction of melanin synthesis on (Fig. 1A). Similar results were obtained assaying basal melanogenesis (Fig. 1B). In the case of MAPKinase inhibitor III, the 20 µM dose was not used due to toxicity incompatible with long-term experiments (Fig. S1).

### Effect of pyridinyl imidazole compounds on melanogenic proteins expression

**Figure 2:**
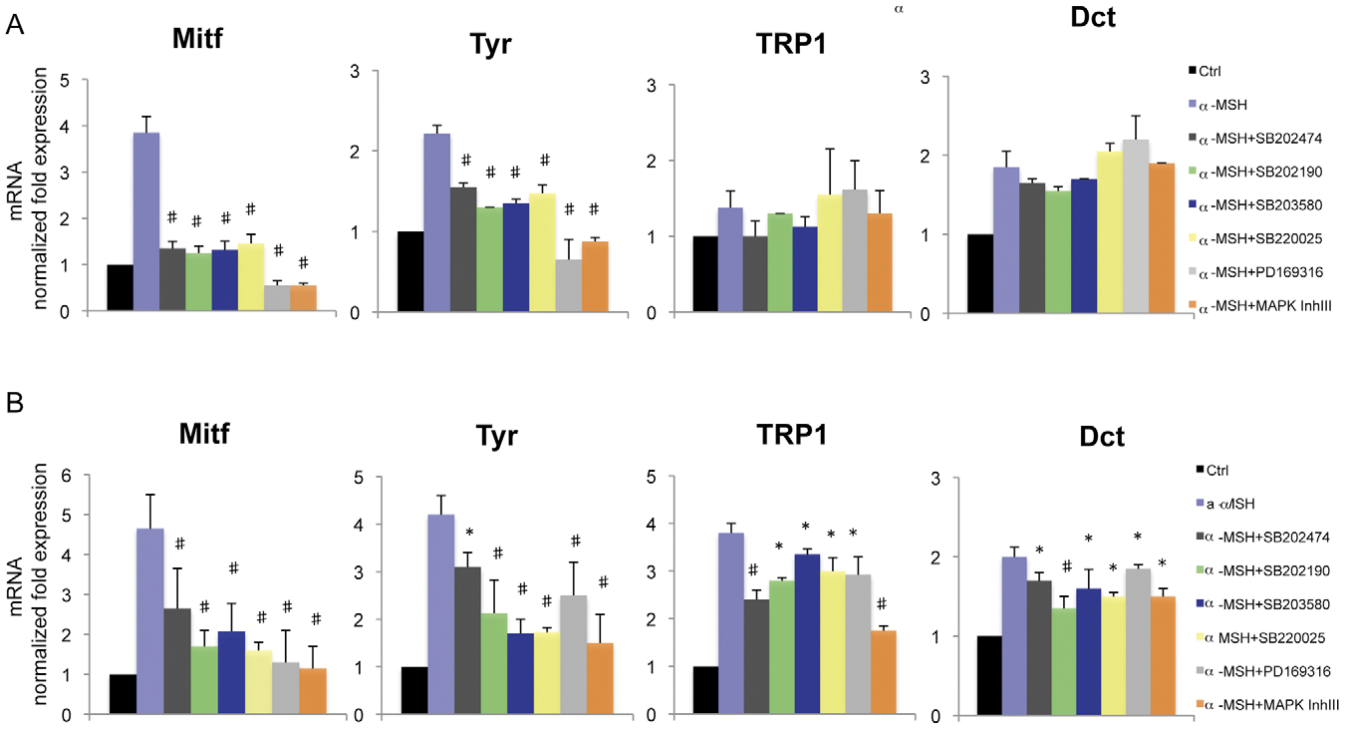
The effect of pyridinyl imidazoles on melanogenic gene expression. (A) Semi-qunatitative real-time PCR was used to measure Mitf, tyrosinase, TRP1 and TRP2 mRNAs expression in B16-F0 cells after 6 h and (B) 24 h of tratments. The graphs show fold differences in transcript abundance in untreated cells, and α-MSH-treated cells (0.1 µM) in presence or not of PI compounds (SB202474, SB202190, SB203580, SB220025, PD169316 20 µM: MAPK Inh III 10 µM). The results shown in (A) and (B) were normalized by the β-actin mRNA levels. The data show the mean±SD of four experiments performed in triplicate. *P≤0.05; #P≤0.01 versus control.

**Figure 3:**
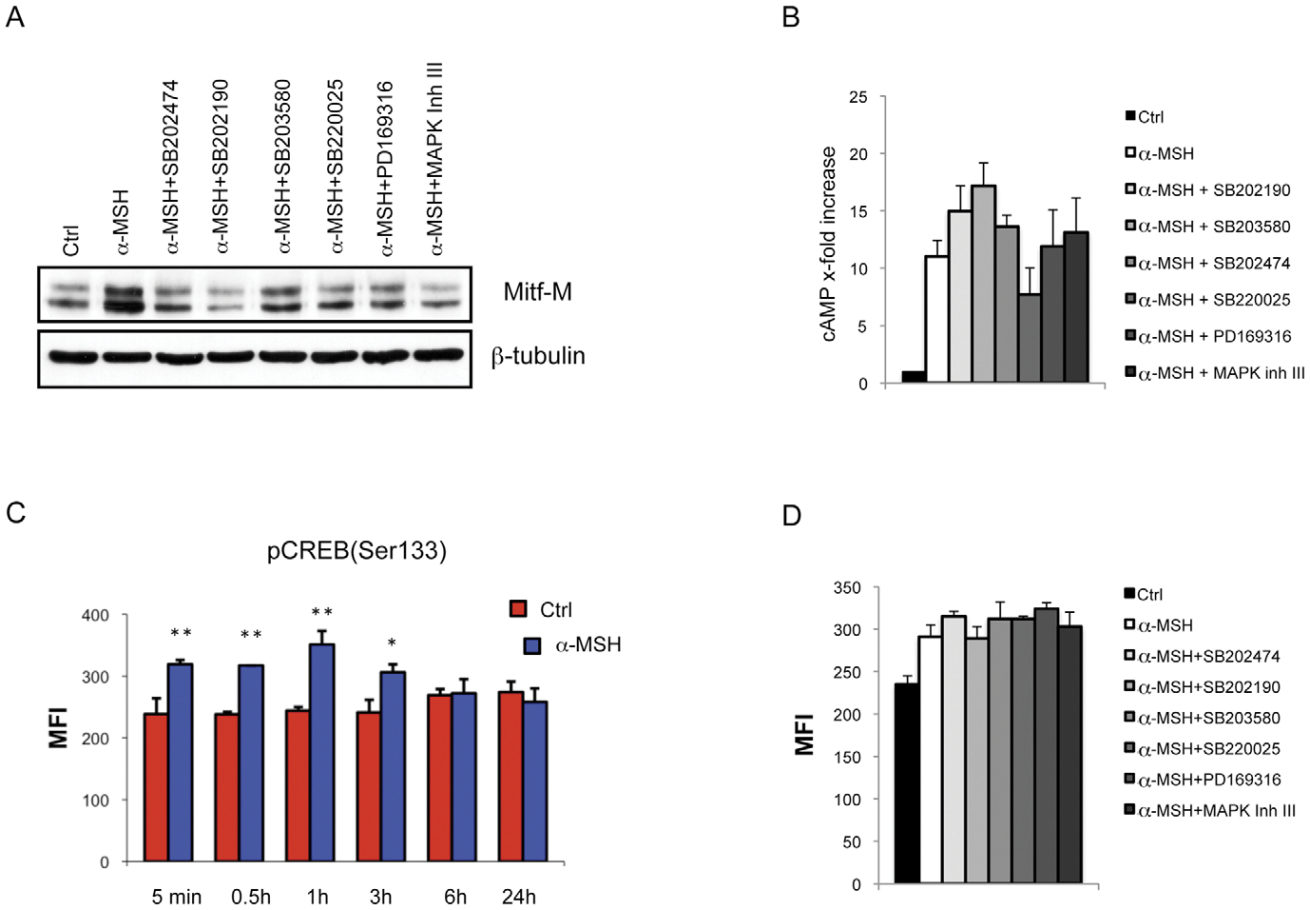
The effect of pyridinyl imidazoles on cAMP/PKA/CREB signal transduction. (A) Expression of Mitf in B16-F0 cells after 6 h of treatment with α-MSH (0.1 µM) in presence or not of PI compounds (SB202474, SB202190, SB203580, SB220025, PD169316 20 µM: MAPK Inh III 10 µM). Total cellular proteins (30 µg/lane) were subject to 10% SDS-PAGE. Variation of loading was determined by blotting with anti-β-tubulin antibody. Western blot assays are representative of at least three experiments. (B) Concentration of cAMP of control and treated cells were determined using the cAMP bioluminescent assay. Following incubation with α-MSH (0.1 µM) in presence or not of PI compounds, the cAMP levels were measured and compared to the untreated control samples. The results are the mean±SD of three experiments performed in duplicates. (C) Analysis of the time-dependent effect α-MSH treatment on CREB level of phosphorylation. Cells were stained with anti-phospho-CREB-PE (Ser133), and then analyzed measuring median fluorescence intensity (MFI) in duplicates. Histogram represents means ± SD of MFI of three independent experimets. (D) Comparative analysis of CREB-Ser133 level of phosphorylation in untreated cells, α-MSH-treated cells and α-MSH plus PI coumpond-treated cells.

**Figure 4:**
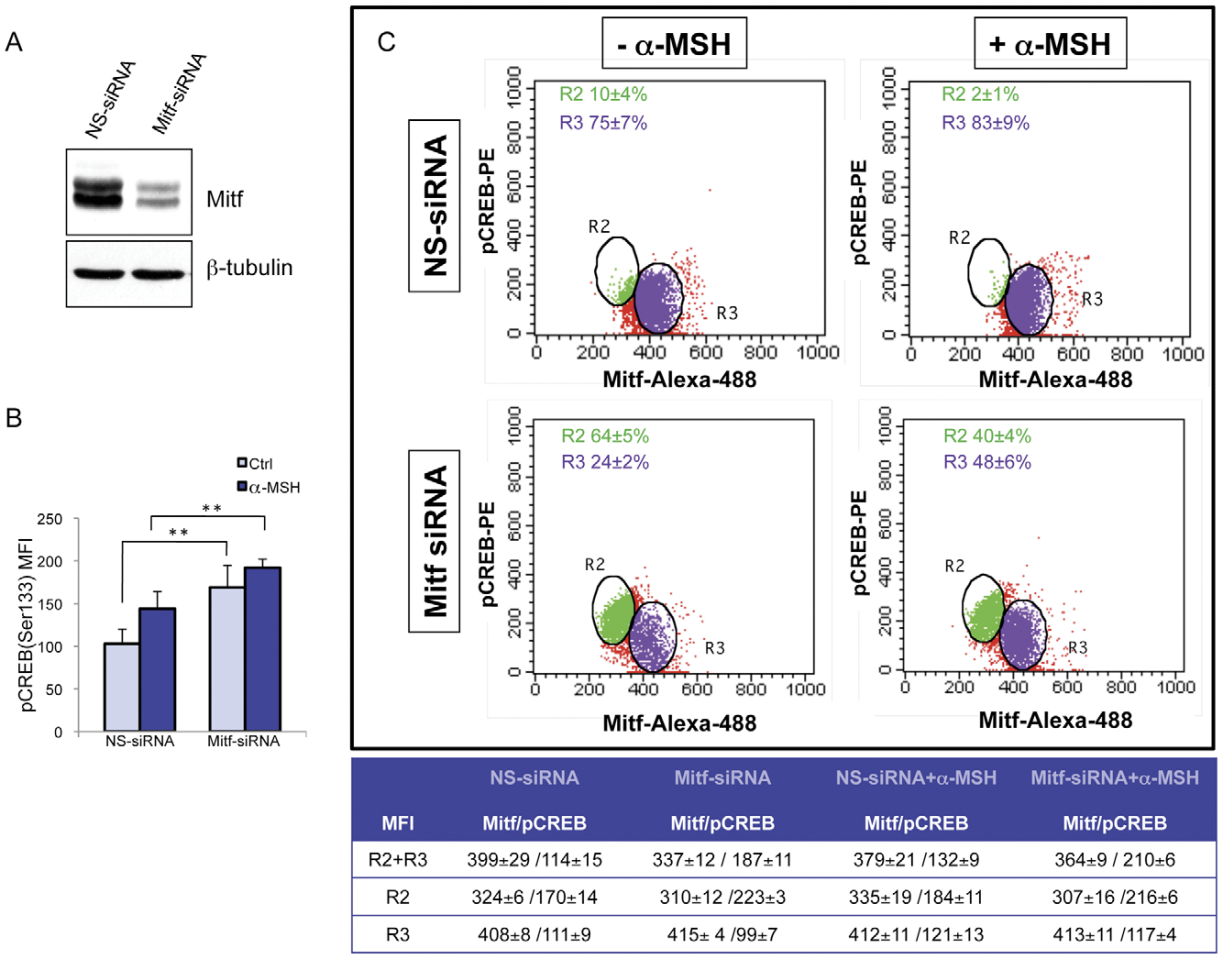
Coordinated regulation of Mitf expression and CREB activation. (A) One representative western blot anti-Mitf used to quantify RNA interference efficiency after 24 h of nucleofection. (B) Analysis of Mitf siRNA interference on CREB level of phosphorylation was analyzed measuring CREB level of phosphorylation by measuring PE-CREB-Ser133 median fluorescence intensity (MFI) in duplicates. Histogram represents means±SD of MFI of three independent experimets. (C) Dot plot analysis of one representative experiments of Mitf-siRNA showing higher MFI of PE-CREB-Ser133 in samples presenting low levels of Mitf expression (region R2) in comparison with the cell fraction presenting high levels of Mitf (region R3).

The regulation of intracellular cAMP levels contributes significantly to both basal and stimulated acquisition of melanocyte differentiation markers [43]. cAMP-induced melanogenesis has been reported to be mediated by the activation of CREB. Transactivation exerted by CREB requires phosphorylation on Ser133 [44] since only the phosphorylated form of CREB is capable of interaction with the transcriptional machinery [45]. Once phosphorylated, CREB can up-regulate Mitf, which in turn transcriptionally activates melanogenic enzymes [12]. Therefore, we examined the effect of pyridinyl imidazole compounds on Mitf and on the three melanocyte-specific enzymes, tyrosinase, TRP1 and Dct mRNA levels after 6 and 24 h of treatment. These experimental time points were chosen because Mitf mRNA increases to its maximal level around 6 h after the addition of α-MSH to the cells, and the maximal change in melanogenic enzyme mRNA abundance is observed at 24 h after stimulation (Fig. S2). The results clearly demonstrated that all PI compounds significantly reduced the production of Mitf and tyrosinase mRNA (Fig. 2A). The transcript levels for both TRP1 and Dct showed little correlation with changes in Mitf expression at 6 hours and changes in the abundance of mRNAs seen with these agents were variable; however, after 24 h of treatment, the analysis of TRP1 and Dct mRNA levels revealed moderate but significant decrease in transcript abundance of both genes (Fig. 2B). Changes in Mitf mRNA expression were reflected by changes in the corresponding protein as determined by western analysis (Fig. 3A). Unlike total protein levels, the ratio of phosphorylated to non-phosphorylated Mitf was retained. Altogether, these results indicated that reduced *Mitf* gene transcription led to consequent effects on melanogenic enzyme production and was responsible for the attenuation of melanogenesis in the presence of PI compounds. As pyrimidinyl imidazole compounds showed an inhibitory effect on Mitf mRNA levels, we also measured the generation of cAMP and the phosphorylation of CREB, two downstream events rapidly generated by the activation of the melanogenic pathway through α-MSH-dependent stimulation of melanocortin 1 receptor (MC1R). The intracellular elevation of cAMP levels was not decreased by co-treatment with pyridinyl imidazole compounds (Fig. 3B). By contrast, we did detect a general slight amplification of intracellular signaling generated by adenylate cyclase activation. The consequent activation of cAMP-dependent protein kinase (PKA) produces a rapid increase in phosphorylated CREB (Ser133) (maximum increase 1.5-fold at 1 h) (Fig. 3C), which then gradually declines to its basal level after 6 hours. Consistent with the elevation of intracellular cAMP, treatment of B16-F0 cells with pyridinyl imidazole compounds in association with α-MSH did not reduce CREB phosphorylation (Fig. 3D). In fact, in the presence of these agents we detected a hyperphosphorylation of CREB that persisted over the normal duration of α-MSH-induced CREB activation (data not shown). Therefore, we concluded that the lack of cAMP-dependent melanogenesis in the presence of pyridinyl imidazole compounds was a CREB phosphorylation-independent event. As the hyperphosphorylation of CREB was an unexpected result, we next evaluated the possibility that CREB hyperphosphorylation is a physiological consequence of Mitf upregulation failure. We tested this hypothesis by utilizing siRNA against Mitf to evaluate the effect of its down-regulation on CREB phosphorylation. Inhibiting Mitf expression (Fig. 4A), even in absence of α-MSH, significantly increased pCREB(Ser-133) levels as measured by median fluorescence intensity (MFI) (Fig. 4B). To confirm whether Mitf down-regulation and CREB phosphorylation occurred concurrently in the same cell populations, we double-stained the cells with anti-Mitf and anti-pCREB(Ser-133) antibodies after 2 hours of α-MSH stimulation (or not) and then performed FACS analysis. The level of CREB phosphorylation was significantly increased in Mitf-siRNA-treated cells (MFI: [R2+R3] NS-siRNA 114±15; Mitf-siRNA 187±11; NS-siRNA+α-MSH 132±9; Mitf-siRNA+α-MSH 166±6). Moreover, the data showed that in all samples the cell fraction presenting low levels of Mitf expression (region R2) contained high levels of CREB phosphorylation in comparison with the cell fraction presenting high levels of Mitf (region R3) (Fig. 4C). According with western blot analysis (Fig. 4A) the percentage of cells with high levels of Mitf expression was lower in Mitf-siRNA treated-cells than in control cells (R3: 24±2% in Mitf-siRNA cells vs 75±7% in NS-siRNA cells; 83±9% NS-siRNA+α-MSH cells vs 48±6% Mitf-siRNA+ α-MSH cells). These results confirmed the existence of regulation coordinating Mitf expression and CREB activation.

### Overexpression of Mitf relieved pyridinyl imidazole compound-mediated repression of melanogenesis

**Figure 5:**
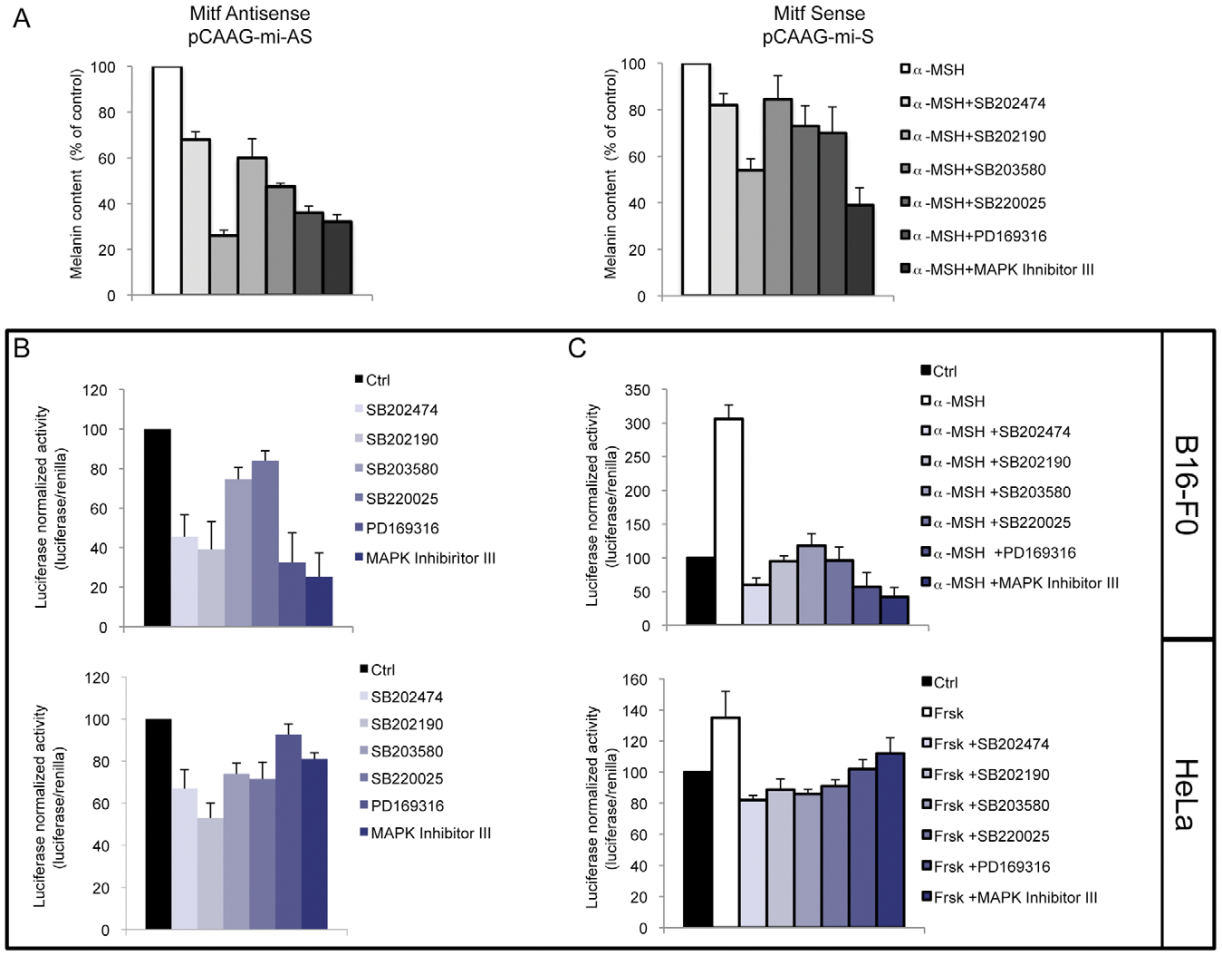
Analysis of the role of Mitf expression in pyridinyl imidazoles-dependent melanogenesis inhibition. (A) To B16-F0 melanoma cells were transiently transfected with a plasmid encoding for Mitf cDNA (pCAAG-mi-S) or a control construct carrying Mitf cDNA in antisense orientation (pCAAG-mi-AS). Following incubation with α-MSH (0.1 µM) in presence of pyridinyl imidazoles (SB202474, SB202190, SB203580, SB220025, PD169316 20 µM: MAPK Inh III 10 µM) for 72 h, or not, the extracellular and intracellular levels of melanin were determined as described above. The data show the mean±SD of three experiments performed in duplicate. (B–C) Analysis of luciferase activity of the Mitf melanocyte-specific promoter (M promoter) in the presence of pyridinyl imidazoles in B16-F0 and HeLa cells. Twenty-four hours after transient transfection cells were treated with PI compounds in presence (C) or not (B) of α-MSH (0.1 µM) (B16-F0) or forskolin (1 µM) (HeLa). Luciferase activity was assayed after 6 h of treatment. Firefly luciferase activity, normalized to the corrisponding renilla luciferase activity was expressed as fold change compared with control cells. Values represent mean ± SD of three representative experiments performed in duplicate.

To ascertain whether decreased Mitf expression could fully explain the depigmentation effects of pyridinyl imidazole compounds, we transiently transfected B16-F0 melanoma cells with a plasmid encoding for Mitf cDNA (pCAAG-mi-S) or a control construct carrying Mitf cDNA in antisense orientation (pCAAG-mi-AS). As shown in Figure 5A, forced exogenous expression of Mitf restores α-MSH-induced stimulation of melanogenesis, confirming that regulation of Mitf expression plays a key role in melanogenesis. Similar results were also obtained assaying basal melanogenesis (data not shown). Thus, we concluded that regulation of Mitf expression is the mechanism responsible for melanogenesis inhibition by pyridinyl imidazole compounds.

In contrast to other Mitf isoforms widely expressed in many cell types, Mitf-M has been established as a specific marker for melanocyte-lineage cells [46]. Mitf transcription from the M-specific promoter is up-regulated via separate *cis*-acting elements of a network of HMG-containing proteins including Sox10, Pax3, and Lef1 (Lef1/β-catenin complex). Moreover, Mitf functions as a non-DNA binding cofactor of Lef1 on its own promoter [47]. To further investigate the regulation of the M promoter in the presence of pyridinyl imidazoles, we transfected a plasmid (pMitf-Luc) containing the Mitf melanocyte-specific promoter sequence (approximately 1 kb) upstream of the luciferase reporter gene (pGL3 basic vector). This construct contains proximal consensus sequences for Sox10, Pax3, Lef1 and CREB. Transient cell transfections were carried out in B16-F0 and in HeLa cells, which were chosen because of their lack of endogenous Mitf [48], Pax3 and Sox10 proteins [49]. As a consequence, the responsiveness of pMitf-Luc in HeLa cells is exclusively dependent on CREB and Lef1 functional activation. Due to the lack of MC1R expression in HeLa cells, in this system CREB activation was achieved via forskolin, an adenylate cyclase activator widely used to stimulate cAMP by an MC1R-independent pathway. All the tested PI compounds repressed luciferase expression in both unstimulated (Fig. 5B) and stimulated cells (Fig. 5C). Treatment with 100 nM α-MSH induced about a 3-fold increase in luciferase activity in transfected B16-F0 cells, whereas 10 µM forskolin stimulated a 1.5-fold increase in luciferase activity in HeLa transfected cells. As expected, luciferase activation was higher in melanoma than in HeLa cells due to the cooperation of melanocyte specific components (Sox10 and Pax3), cAMP signaling machinery and the physiological tissue-specific expression of the M-Mitf promoter. However, the overall inhibition caused by imidazoles treatment was similar in both cell lines, suggesting that the interference of these compounds with cAMP responsiveness of Mitf promoter is direct to CREB and/or Lef1 transcription factors functionality.

### Pyridinyl imidazole compounds down-regulate melanogenesis via suppression of Wnt/β-catenin signaling

**Figure 6:**
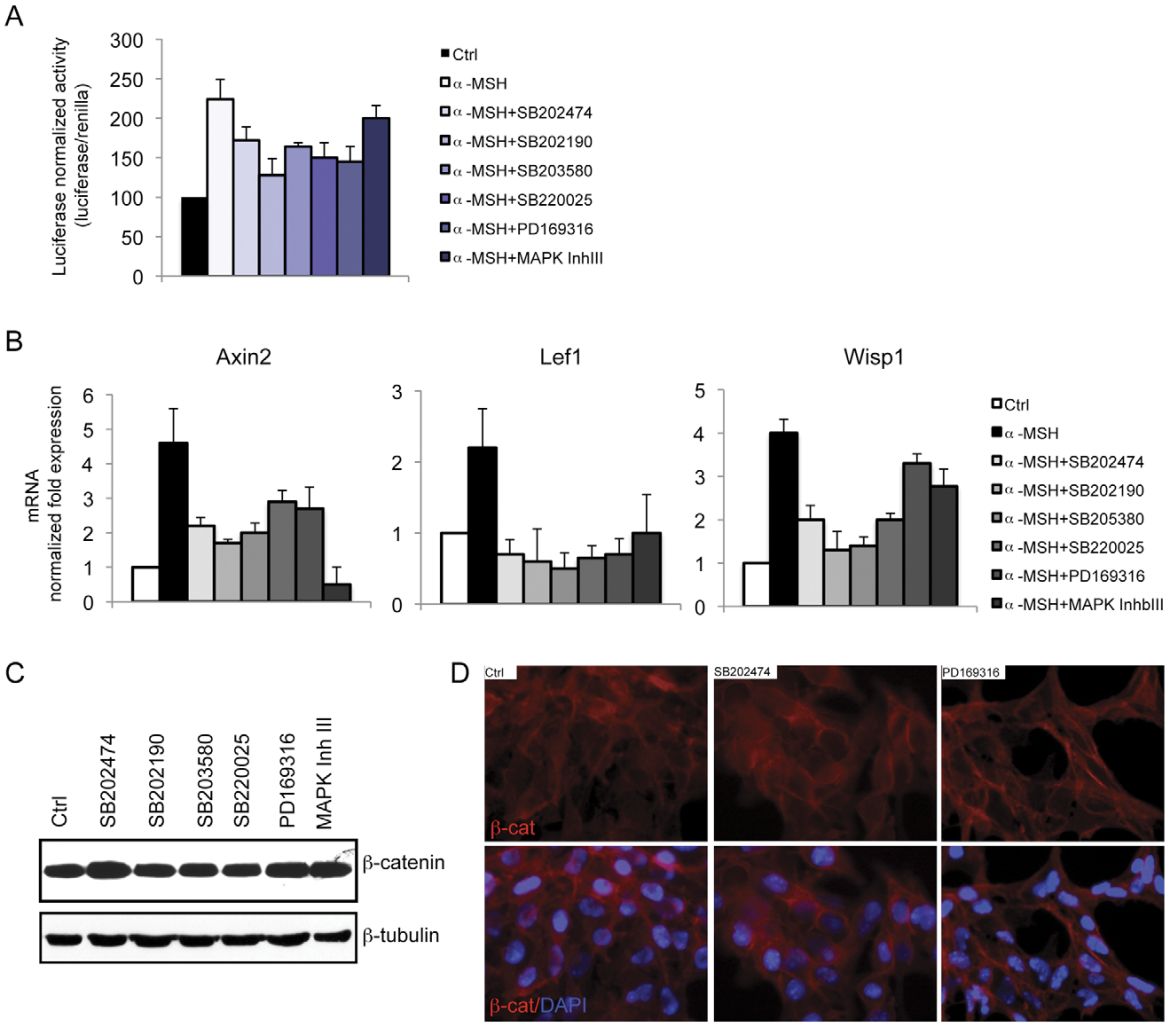
Regulation of Wnt/β-catenin signaling by pyridinyl imidazoles. (A) Inhibition of the β-catenin/Tcf/Lef1-responsive luciferase reporter gene by PI compounds. The pTK-Renilla was inserted as an internal control. Twenty-four hours after transfection, cells were treated with PI compounds (SB202474, SB202190, SB203580, SB220025, PD169316 20 µM: MAPK Inh III 10 µM) for 6 h. Firefly luciferase activity, normalized to the corresponding renilla luciferase activity was expressed as fold decrease compared with control cells. Values represent mean ± SD of three representative experiments performed in duplicate. (B) Semi-qunatitative real-time PCR was used to measure Wnt/β-catenin-target genes Axin2, Lef1 and Wisp1 mRNAs expression in B16-F0 cells after 6 h of treatments with PI compounds. The graphs show fold differences in transcript abundance in comparison with untreated cells. Results shown were normalized by the β-actin mRNA levels. The data show the mean±SD of three experiments performed in triplicate. *P≤0.05; #P≤0.01 versus control. (C) Expression of β-catenin in B16-F0 cells after 6 h of treatment with PI compounds (SB202474, SB202190, SB203580, SB220025, PD169316 20 µM: MAPK Inh III 10 µM). Total cellular proteins (30 µg/lane) were subject to 10% SDS-PAGE. Variation of loading was determined by blotting with anti-β-tubulin antibody. Western blot assays are representative of at least three experiments. (D) Immunofluorescence analysis of β-catenin. B16-F0 cells were grown on glass coverslips and then treated with SB202474, PD169316 (20 µM) or DMSO respectively. Six hours later, cells were fixed and analyzed by immunofluorescence labelling with a mouse monoclonal anti-β-catenin followed by Alexa-Fluor-546-conjugated goat anti-mouse IgG antibody. Nuclei were labelled with bisbenzidine (DAPI). Original magnification 20×.

**Figure 7:**
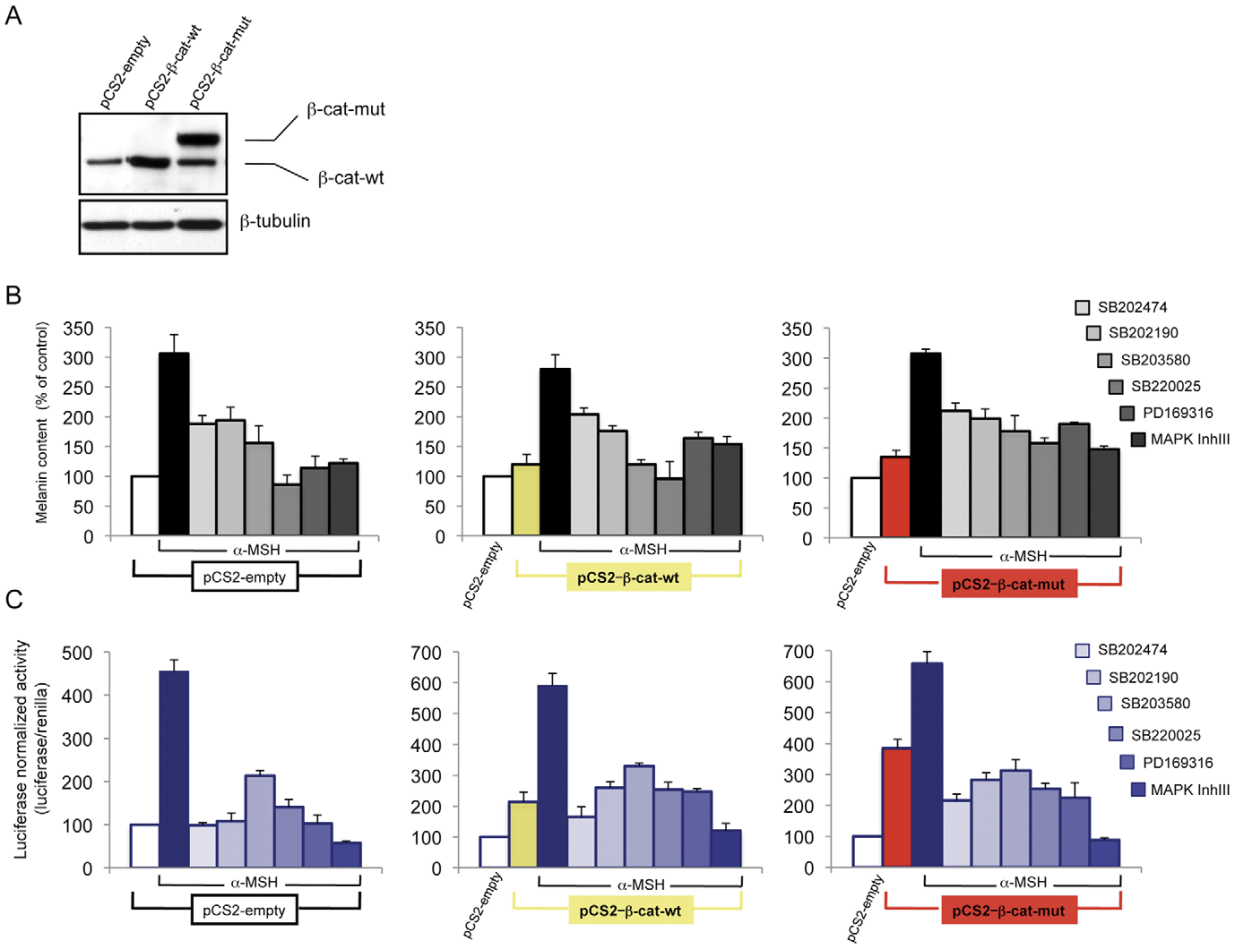
The effect of forced β-catenin expression on pyridinyl imidazoles-dependent melanogenesis inhibition. Western blot analysis of β-catenin in whole-cell lysates prepared from B16-F0 cells transfected with pCS2-β-cat-wt, pCS2-β-cat-mut plasmids or pCS2 empty vector. Total cellular proteins (30 µg/lane) were subject to 10% SDS-PAGE. Variation of loading was determined by blotting with anti-β-tubulin antibody. Western blot assays are representative of at least three experiments. (B) Twenty-four hours after transfection cells were treated with α-MSH (0.1 µM) in presence of pyridinyl imidazoles (SB202474, SB202190, SB203580, SB220025, PD169316 20 µM: MAPK Inh III 10 µM) for 72 h, or not, and then the extracellular and intracellular levels of melanin were determined as described above. The data show the mean±SD of three independent experiments performed in duplicate. (C) B16-F0 cells were cotrasfected with lucerase reporter plasmids TopFlash and pCS2-β-cat-wt, pCS2-β-cat-mut plasmids. The pTK-Renilla was inserted as an internal control for each experimental sample as a control for transfection efficiency. Firefly luciferase activity, normalized to the renilla luciferase activity was expressed as fold decrease compared with control cells. Values represent mean±SD of three representative experiments performed in duplicate.

Previous studies have demonstrated that Wnt/β-catenin signaling controls melanocyte differentiation primarily through the direct regulation of Mitf [50]. We recently reported that melanocortin-stimulated melanocyte differentiation up-regulates β-catenin transcriptional activity and that cooperation between CREB and β-catenin on the Mitf-M promoter is essential for realization of the melanogenic program [15]. Given that pyridinyl imidazole compounds repressed Mitf expression in the absence of any evident CREB activation defect, we determined whether the repression of melanogenesis observed in the presence of pyridinyl imidazole compounds could be mediated by an antagonist effect on Wnt/β-catenin signaling. In B16-F0 cells transfected with the TopFlash (Tcf/Lef1) reporter plasmid, α-MSH-induced activity was significantly reduced by the presence of pyridinyl imidazole compounds (Fig. 6A). As the TopFlash plasmid contains a synthetic promoter, we also tested the expression of genes that are naturally stimulated by the β-catenin/Tcf/Lef1 complex. The expression of Axin2, Lef1 and Wisp1, which we previously demonstrated to be significantly stimulated by intracellular cAMP elevation, was dramatically reduced in the presence of pyridinyl imidazoles (Fig. 6B). Western blot and immunofluorescence analysis, however, did not show any decrease in β-catenin protein expression (Fig. 6C and D). These results indicate that the functional inhibition of β-catenin activity may be dominant over the small protein increase. Accordingly, forced over-expression of either wild type or a constitutively active mutant form of β-catenin that cannot be phosphorylated by CKIα and GSK3β (Fig. 7A) moderately counteracted pyridinyl imidazole-dependent repression of melanogenesis and inhibition of pMitf-luciferase activity (Fig. 7B and C). Similar results were obtained in absence of hormonal stimulation measuring the basal level of melanin synthesis (data not shown). This result confirms that the regulation of β-catenin-dependent transcriptional activity and not simply β-catenin protein levels makes an important contribution to melanin synthesis.

**Figure 8:**
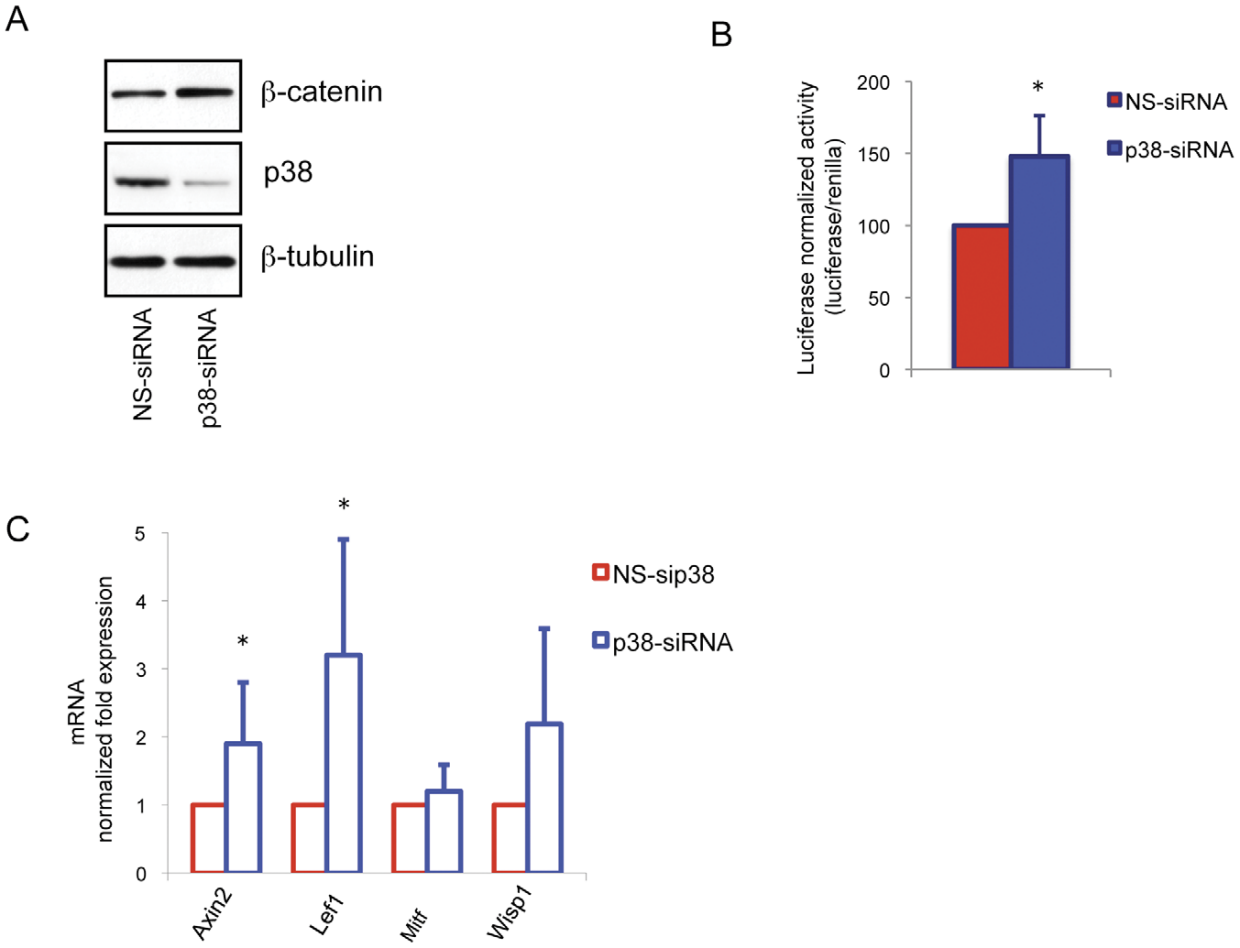
The effect of p38 silencing on Wnt/β-catenin pathway signal transduction. (A) One representative western blot used to quantify p38-siRNA interference efficiency and β-catenin 24 h of transfection. Total cellular proteins (20 µg/lane) were subject to 10% SDS-PAGE. Variation of loading was determined by blotting with anti-β-tubulin antibody. Western blot assays are representative of at least three experiments. (B) Luciferase activity of TopFlash reporter plasmid in cells transfected with p38-siRNA and NS-siRNA. Luciferase activity was assayed after 24 h of transfection. Firefly luciferase activity, normalized to the corrisponding renilla luciferase activity was expressed as fold change compared with control cells. Values represent mean±SD of three representative experiments performed in duplicate. (C) Semi-qunatitative real-time PCR was used to measure Wnt/β-catenin-target genes Axin2, Lef1 and Wisp1 mRNAs expression in B16-F0 cells after 24 h of transfection with p38-siRNA. The graphs show fold differences in transcript abundance in comparison with non-specific siRNA-treated cells. Results shown were normalized by the β-actin mRNA levels. The data show the mean±SD of three experiments performed in triplicate. *P≤0.05 versus control.

A possible intersection of the MAPK pathway with the Wnt/β-catenin signaling pathway has been suggested in mouse F9 teratocarcinoma cells and HEK293 human embryonic cells [51], [52]. However, a more recent study demonstrated that the knockdown of p38 does not interfere with Wnt/β-catenin signal transduction and suggested that small molecule p38 MAPK inhibitors may have cross-reactivity between these two pathways [53]. To evaluate the possible impact of p38-reduced activity on β-catenin–dependent transcription in B16-F0 cells were transiently transfected with siRNA against p38 or a non-specific control (NS-siRNA). Samples treated with p38 siRNA showed a moderate increased expression and functional activation of β-catenin as demonstrated by western blot analysis, TopFlash luciferase activity and stimulation of Wnt/β-catenin target genes expression (Fig. 8A, B and C). These results conclusively show that these small molecules possess two distinct and additive ways to modulate β-catenin dependent transcription: a specific p38 inhibition-dependent mechanism stimulating the pathway (by increasing β-catenin protein expression) and an off-target mechanism inhibiting the pathway (by repressing β-catenin protein functionality). The p38-indendent effect seems to be dominant, and at least in B16-F0 cells, the final outcome is a strong block of the Wnt/β-catenin signaling pathway.

## Discussion

Previous studies demonstrated that despite being used widely, the pyridinyl imidazole class of p38 MAPK inhibitors as well as the inactive compound SB202474 are involved in many different p38-independent cellular activities, such as cyclic nucleotide accumulation [54], nucleoside transport [37], aryl hydrocarbon receptor target gene activation [38], defective autophagic vacuole formation [38] and CKI kinase activity regulation [39], [53]. Compounds analyzed in the present study were found to be too non-specific to assess the physiological roles of p38 MAPK in melanocyte differentiation. Our preliminary results demonstrated that the p38 inhibitors (SB202190 and SB203580) and the structural analog inactive compound SB202474 were effective in reducing melanogenesis suggesting that this effect is likely due to pharmacological actions of these compounds that are unrelated to their inhibition of p38 MAPK activity. This hypothesis is also supported by the observation that p38 MAPK specific siRNA stimulates melanogenesis [42]. In line with the concept that the observed inhibition is p38-independent, we observed that the efficiencies of these compounds in melanogenesis inhibition were not consistent with published IC_50_ values against p38 MAP kinase activity (see table S1). For example, MAPK inhibitor III (IC_50_ = 380 nM) and PD169316 (IC_50_ = 89 nM) at doses of 10 and 5 µM reduced melanin synthesis more strongly than SB202190 (IC_50_ = 50 nM) and SB220025 (IC_50_ = 60 nM).

In this study, all pyridinyl imidazole compounds tested suppressed melanin synthesis as well as the expression of Mitf, and consequently TRP1 and Dct, by blocking β-catenin-dependent transcription. The centrality of Mitf protein regulation in imidazole-dependent melanogenesis inhibition was also confirmed by transfection experiments that clearly demonstrated restoration of melanin synthesis by forced exogenous Mitf expression. Four transcription factors are known that regulate expression of the melanocyte-specific Mitf-M isoforms: Sox10, Pax3, CREB and Lef1. Data obtained from HeLa cells indicate that imidazoles act on ubiquitous factors, such as CREB, Lef1, and related Tcf factors more than on melanocyte-specific proteins such as Sox10 and Pax3. Moreover, the level of cAMP as well as the hyperphosphorylation of CREB suggested a compensatory mechanism trigged by Mitf expression failure, excluding a defect in the cAMP/PKA/CREB axis responsiveness. Based on these results, we restricted our investigation to Lef1. Lef1 transcription factor is responsive to Wnt signaling through its interaction with β-catenin [18]. Imidazole-treated cells showed a reduction in the level of Tcf/Lef targets involved in the Wnt signaling network, including ubiquitous genes such as Axin2, Lef1, and Wisp1 as well as cell lineage-restricted genes such as Mitf and Dct. Although the over-expression of the Wnt signaling pathway effector β-catenin partially recovered the melanogenic program, the lack of complete reversion suggested that imidazole interferes with β-catenin-dependent transcriptional activity rather than simply affecting β-catenin protein expression. Accordingly, we did not observe any significant decrease of β-catenin protein expression. Therefore, in agree with results obtained with p38-siRNA experiments a slight increase of β-catenin level of expression was evident, especially in presence of PD169316 and MAPK inhibitor III. This result could also be explained by a p38-independent-mechanism since SB202190 and SB203580 also inhibits, at least *in vitro*, GSK3β activity [55].

It is becoming increasingly clear that a variety of routes exist that modulate the Wnt/β-catenin pathway and that β-catenin signaling regulation may be more complicated than the simplified view of β-catenin cellular homeostasis. Accordingly, we previously demonstrated that over-expression of β-catenin slightly activates Tcf/Lef-mediated transcription in melanocyte lineages [15] but is not sufficient to prime the melanogenic program [56], suggesting that even if β-catenin could synergize with other pathways the exclusive presence of a large amount of nuclear β-catenin is not sufficient to activate the melanogenic program. Moreover, a large body of evidence demonstrates that β-catenin binds the two closely related transcriptional co-activating proteins p300 and CBP in a mutually exclusive fashion, and as a result, different subsets of genes can be expressed [57]. Interestingly, the choice of co-activator can be redirected by small compounds independently of the level of β-catenin expression [28], [58]. Furthermore, inhibition of the histone acetyltransferase activity of basic transcription factors has been proposed as a possible mechanism of pyridinyl imidazoles-dependent suppression of gene transcription induced by aromatic hydrocarbons [59]. Moreover, as pyrrole-imidazole polyamides are able to inhibit Lef1-DNA binding and Lef1-activated transcription both *in vitro* [60] and in cultured cells [61], we cannot exclude the possibility that the aromatic rings of the *N*-methylpyrrole and *N*-methylimidazole amino acid contained in pyridinyl imidazole derivatives suppress the recruitment of Lef1 or other co-activators on Wnt target gene promoters. Further studies to elucidate the exact mechanism of action of pyridinyl imidazoles are currently on-going in our laboratory.

In this paper, we described possible mechanisms by which pyridinyl imidazole derivates may suppress melanin synthesis by acting on Wnt signaling through unknown target molecules. Nevertheless, it is evident that the PI-dependent interference with Wnt signaling leads to low level of Mitf expression. However, is it also important to consider that the decreased Lef1 expression could trigger a negative loop reinforcing the inhibition of β-catenin-dependent genes transcription. Importantly, attenuation of Wnt signaling and decreased level of melanin synthesis were also confirmed in normal human epidermal melanocyte cell cultures (data not shown) supporting a possible topical application to treat disorders of hyperpigmentation. A prerequisite of further exploring the possibility of using PI compounds as skin-whitening agents is the knowledge of any additional effects. For example, we observed that while SB202474, SB202190, SB203580, PD169319 and MAPK inhibitor III all caused a strong p38-independent retention of melanin in the intracellular compartment, SB220025 did not alter melanin secretion (data not shown).

From the therapeutic point of view, there is an increasing interest in small molecules capable of regulating the Wnt signaling cascade as this pathway is involved in cancer cell proliferation and migration. In addition, reduction of Mitf activity sensitizes melanoma, a highly chemotherapy-resistant neoplasm, to chemotherapeutic agents, and it has been suggested that targeting Mitf in combination with B-RAF or cyclin dependent kinases inhibitors may offer a rational therapeutic avenue into melanoma [62]. Thus, a clinical evaluation of pyridinyl imidazole compounds targeting Wnt/β-catenin in the absence of p38 MAPK cross-reactivity (such as the case of SB202474) could be of extreme interest.

## Materials and Methods

### Cell culture transfection and reagents

The B16-F0 murine melanoma and HeLa cells were purchased from ATCC (American Type Culture Collection) and were maintained in Dulbecco's modified Eagle's medium (DMEM)+7% (B16-F0) or 10% (HeLa) heat-inactivated fetal bovine serum (FBS) and antibiotics (Gibco, Life Technologies Italia, Milan, Italy). For the induction studies, cells were plated for 24 hours, and then the medium was removed and the cells cultured in DMEM with 2% heat-inactivated FBS and antibiotics with or without pharmacological treatment in the absence of phenol red. SB202190, SB203580, α-MSH, DMSO and L-DOPA were purchased from Sigma (Sigma Aldrich, Milan, Italy), while MAPK inhibitor III, PD169316, SB202474, and SB220025 were purchased from Calbiochem (Merck, Milan, Italy). For transient transfection experiments cells, 1.5×10^6^, were transfected with Amaxa Nucleofector System using Amaxa Nucleofector Cell Line Kit R (program P-031). Cells were transfected by nucleofection in a single cuvette and plated immediately to ensure identical transfection efficiency. For siRNA experiments, we transferred 200 pmols of either p38 specificsiRNA, Mitf specific siRNA or scrambled siRNA as a negative control. Twenty-four hours later, cells were treated with compounds or DMSO. For Mitf or β-catenin (wild-type or mutated) exogenous over-expression cells, 1.5×10^6^, were transfected with 5 µg of the corresponding construct using Amaxa Nucleofector System. For luciferase experiments cells, 1.5×10^6^, were transfected with 1 µg of p-Mitf-Luc containing the Mitf melanocyte-specific promoter sequence upstream of the luciferase reporter gene or pTopFlash luciferase reporter plasmids containing three copies of the optimal Tcf/Lef-binding site (AAGATCAAAGGGGGT) upstream of a TK minimal promoter. A pTK-Renilla–expressing vector (Promega, Milan, Italy) was included as an internal control to avoid non-specific effects on luciferase expression treatment and to normalize data. After 24 hours, cells were treated with compounds (or not) and 6 hours later cells were lyzed and resuspended in reporter lysis buffer. Firefly and Renilla luciferase activity were determined with Dual-Luciferase reporter assay (Promega, Milan, Italy) from duplicate plates. The results represent data of duplicates from three independent experiments.

### Cell viability analysis

Cells were plated in a 24-well plate at a density of 2×10^4^ cells/cm^2^ and left to grow overnight. Cells were treated with increasing concentrations of PI compounds in DMEM containing 2% FBS and antibiotics in quadruplicate, and were left to grow for 72 h before being incubated with 3-(4,5 dimethylthiazol)-2,5-diphenyl tetrazolium bromide (MTT) for 60 min. After this time, the medium was removed and the resulting crystals were solubilized in DMSO. The absorbance was measured at 570 nm with a reference wavelength of 650 nm. Absorbance readings were subtracted from the value of blank wells, and the reduction in cell growth was calculated as a percentage of control absorbance in the absence of any drug (DMSO).

### Melanin content determination

Extracellular melanin release was measured as previously described [42]. Briefly, 200 µl of the media was removed and the absorbance was measured spectrophotometrically at 405 nm using a plate reader to measure extracellular melanin. After extraction of the protein fraction, cell pellets were dissolved in 200 µl of 1 M NaOH for 2 h at 60°C and the absorbance was measured spectrophotometrically at 405 nm using a plate reader. Standard curves using synthetic melanin (0–250 µg/ml) were prepared for each experiment. Melanin production was calculated by normalizing the total melanin values with protein content (µg melanin/mg protein).

### Western Blot analysis

Proteins extracts were prepared with RIPA buffer (Tris-buffered saline, 0.5% deoxycholate, 0.1% SDS, 1% Triton X-100) containing Complete Mini protease inhibitor cocktail (Roche Diagnostic, Milan, Italy). Aliquots of cell lysates were separated by electrophoresis on SDS-polyacrylamide gels, transferred to nitrocellulose membranes and then treated with the appropriate antibodies: anti-β-catenin 1∶3000, anti-Mitf 1∶500, anti-p38 1∶500 (Santa Cruz Biotechnology Inc., Santa Cruz, CA, USA) and anti-tubulin 1∶5000 (Sigma Aldrich, Milan, Italy). Horseradish peroxidase-conjugated goat anti-mouse and bovine anti-goat immunoglobulin (Santa Cruz Biotechnology Inc., Santa Cruz, CA, USA) were used at 1∶5,000 and 1∶3,000, respectively. Antibody complexes were detected by chemiluminescence (ECL; Amersham Life Science, Arlington Heights, IL, USA). Western blot assays were representative of at least three experiments. Densitometric analysis was performed using a GS-800 Calibrated Image Densitometer (BioRad Laboratoires, Milan, Italy).

### Quantitative RT-PCR

Total RNA was extracted using an RNeasy mini kit (Qiagen, Inc., Valencia, CA, USA). cDNA was synthesized from 1 µg of total RNA using the Improm II™ Reverse Transcription System (Promega, Milan, Italy). First-strand cDNA (1 µl) was amplified in a reaction mixture (15 µl) containing BioRad Master SYBR Green and 25 pmol of forward and reverse primers. All samples were run in triplicate, and the average was then used to calculate the C_t_ value of each particular sample. The median ΔC_t_ value, calculated as the differences between the C_t_ value for the gene of interest and that for the endogenous control β-actin, was used to calculate ΔΔC_t_ = ΔC_t treated sample_−ΔC_t control sample_, and this, in turn, was used to calculate the fold difference of the genes of interest between treated and control samples: Normalized expression ratio = 2^−(ΔΔCt)^. Values represent the means±SD of normalized fold-difference (increase or decrease). Data were analyzed with iQ5 Optical System Software (BioRad Laboratories). Sequences of oligonucleotide primers indicated with an F correspond to sense strands and with an R correspond to anti-sense. In preliminary tests the amplification efficiency of all primer pair was showed to be ≥99.5%. Reverse primers can be found in Table S2.

### Flow cytometry

Cells were fixed and permeabilized with Cytofix/Citoperm™. Cells were stained with anti-Mitf (Santa Cruz Biotechology Inc., Santa Cruz, CA, USA) primary antibody followed by an incubation step with goat-anti-mouse-Alexa488 secondary antibody; anti-CREB-PE-conjugated (pSer133) antibody (BD Bioscience, Erembodegem, Belgium) and then analyzed by flow cytometry using a FACSCalibur. Experiments included the following negative controls (no antibody) to confirm staining specificity for single color analysis with anti-CREB-PE-conjugated (pSer133) antibody; goat-anti-mouse-Alexa488 and goat-anti-mouse-Alexa488 plus CREB-PE-conjugated (pSer133) for in double staining experiments. Data from 1×10^4^ cells were acquired from each sample. Median Fluorescence Intensity (MFI) was evaluated on a linear scale.

### Immunofluorescence

For indirect immunofluorescence experiments, cells were grown on coverslips and after treatment were fixed with 3% paraformaldehyde in PBS for 15 min at room temperature and then permeabilized with 0.05% Trition X-100 in PBS for 5 min. Cells were rinsed in PBS and incubated for 2 h with mouse anti-β-catenin (Santa Cruz Biotechnology, Inc., Santa Cruz, CA, USA) primary antibody (1∶1000 in PBS). Cells were rinsed three times with PBS and incubated for 60 min with an Alexa-Fluor-488-conjugated goat anti-mouse IgG (1∶800 in PBS) (Molecular Probe). To confirm staining specificy each experiment included a negative control incubated exclusively with Alexa-Fluor-488-conjugated goat anti-mouse IgG in absence of no primary antibody. Nuclei were stained with DAPI (Sigma, Milan, Italy). Images were captured using a CCD camera (Zeiss, Oberkochen, Germany).

### Statistical analysis

Student's *t*-test was used to assess statistical significance with thresholds of * p≤0.05 and # p≤0.01.

## Supporting Information

Figure S1 **Effect of pyridinyl imidazoles on cell viability.** Cells were left to grow for 72 in presence of increasing concentrations of pyridinyl imidazoles compounds before being incubated with 3-(4,5 dimethylthiazol)-2,5-diphenyl tetrazolium bromide (MTT) for 2 hrs. The resulting crystals were solubilized in DMSO. The absorbance was measured at 570 nm with a reference wavelength of 650 nm. Values reported as O.D. decrease over untreated control represent the means±SD of two experiments performed in triplicate.
(TIF)

Figure S2 **Time-dependent α-MSH-dependent stimulation of Mitf and melanogenic enzymes.** Semi-quantitative real-time-PCR to measure the kinetics of Mitf, tyrosinase, TRP1 and DCT mRNA increase following α-MSH treatment was performed by using the real-time detection system. The graphs show fold differences in transcripts abundance in α-MSH-stimulated cells compared with untreated cells. The results shown were normalized by the β-actin mRNA levels. The data show the mean±SD of four experiments performed in triplicate.
(TIF)

Table S1 **Pyridinyl Imidazole Compounds.** All PI included in the study are listed with the corresponding chemical name and IC_50_.
(DOCX)

Table S2 **Sequences real-time PCR oligonucleotide primers list.**
(DOCX)
