# Supplementary figures and images for: Inhibition of Melanogenesis by the Pyridinyl Imidazole Class of Compounds: Possible Involvement of the Wnt/β-Catenin Signaling Pathway
Source: PLoS One. 2012 Mar 13;7(3):e33021. doi: 10.1371/journal.pone.0033021 (PMC3302780; doi:10.1371/journal.pone.0033021)

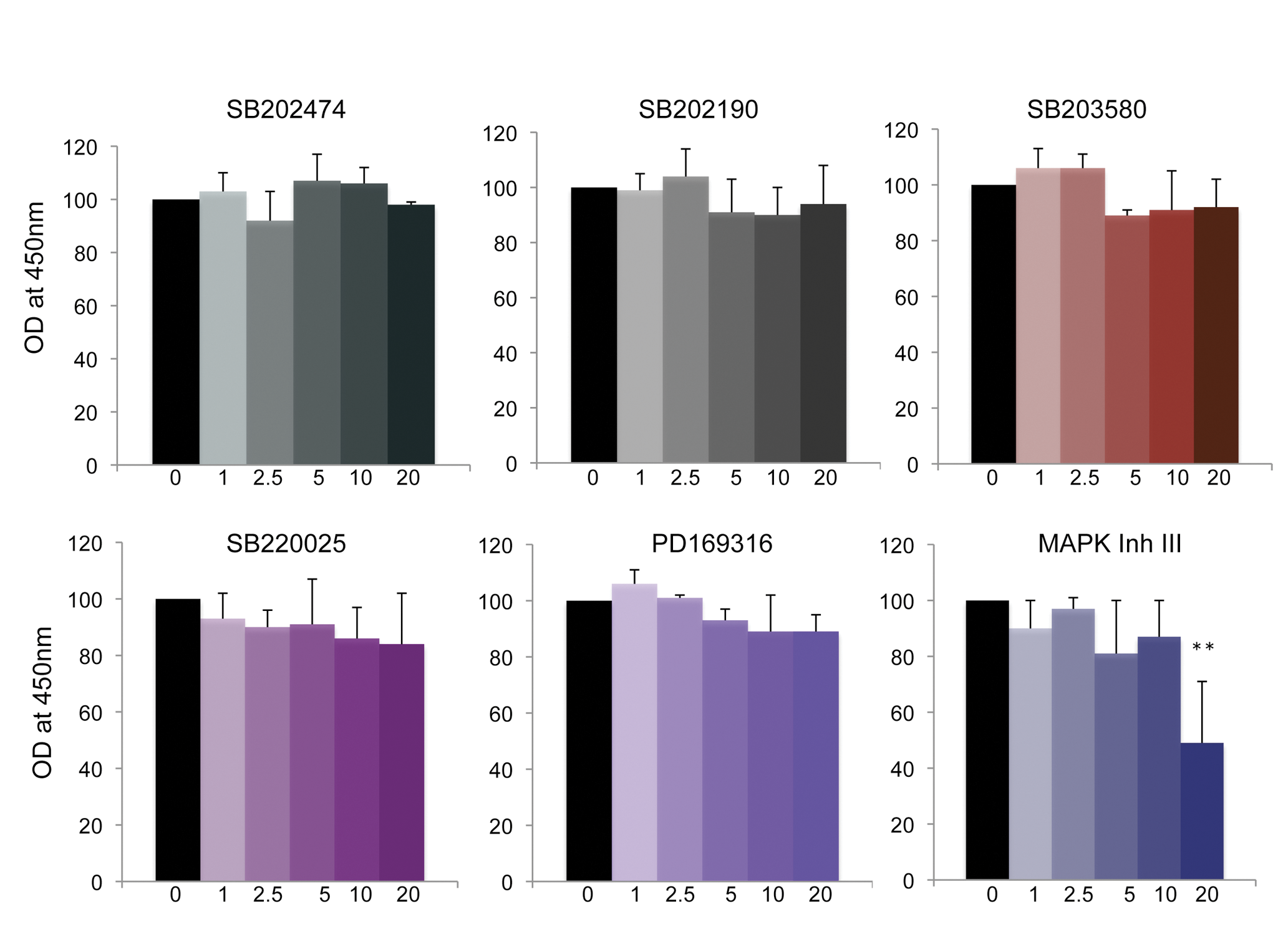

Supplement: Figure S1 — Effect of pyridinyl imidazoles on cell viability. Cells were left to grow for 72 in presence of increasing concentrations of pyridinyl imidazoles compounds before being incubated with 3-(4,5 dimethylthiazol)-2,5-diphenyl tetrazolium bromide (MTT) for 2 hrs. The resulting crystals were solubilized in DMSO. The absorbance was measured at 570 nm with a reference wavelength of 650 nm. Values reported as O.D. decrease over untreated control represent the means±SD of two experiments performed in triplicate. (TIF) [file pone.0033021.s001.tif]

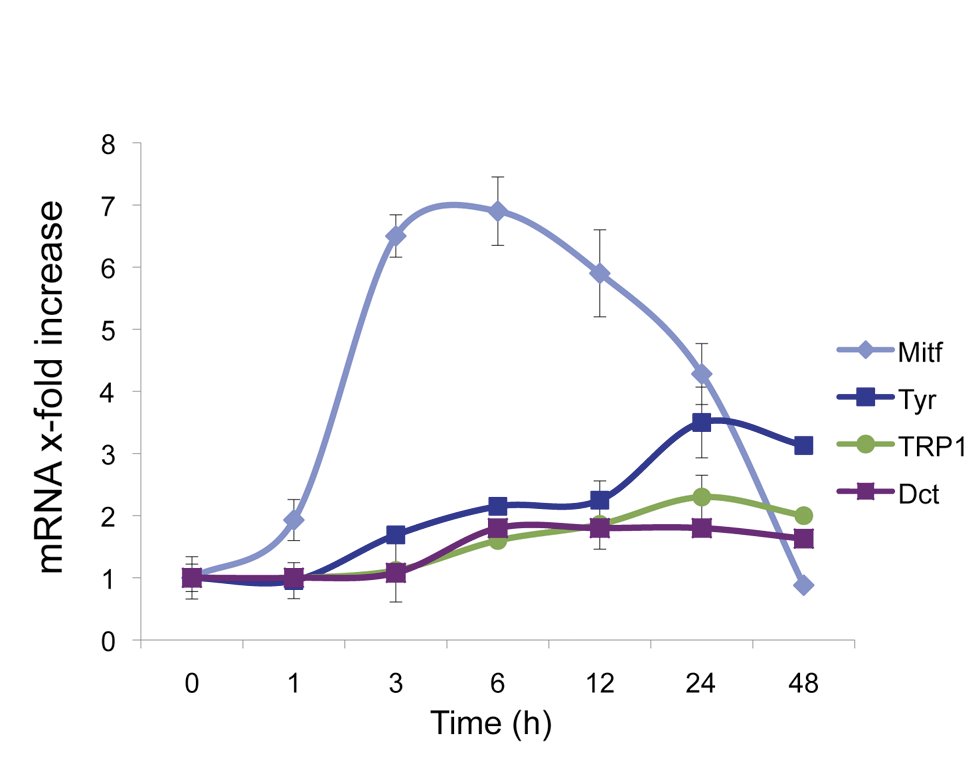

Supplement: Figure S2 — Time-dependent α-MSH-dependent stimulation of Mitf and melanogenic enzymes. Semi-quantitative real-time-PCR to measure the kinetics of Mitf, tyrosinase, TRP1 and DCT mRNA increase following α-MSH treatment was performed by using the real-time detection system. The graphs show fold differences in transcripts abundance in α-MSH-stimulated cells compared with untreated cells. The results shown were normalized by the β-actin mRNA levels. The data show the mean±SD of four experiments performed in triplicate. (TIF) [file pone.0033021.s002.tif]
