# Supplementary material for: Inhibition of Melanogenesis by the Pyridinyl Imidazole Class of Compounds: Possible Involvement of the Wnt/β-Catenin Signaling Pathway
Source: PLoS One. 2012 Mar 13;7(3):e33021. doi: 10.1371/journal.pone.0033021 (PMC3302780; doi:10.1371/journal.pone.0033021)
Supplement: Table S1 — Pyridinyl Imidazole Compounds. All PI included in the study are listed with the corresponding chemical name and IC50. (DOCX) [file pone.0033021.s003.docx]

**Table S1.** Pyridinyl imidazole compounds.

| **Compound** | **In vitro IC50** | **Chemical name** |
| --- | --- | --- |
| SB202474 | Not detected | 4-Ethyl-2(p-methoxyphenyl)-5-(4’-pyridyl)-1H-imidazole |
| SB202190 | 30 nM | 4-(4-Fluorophenyl)-2-(4-hydroxyphenyl)-5-(4-pyridyl)1H-imidazole |
| SB203580 | 34 nM | 4-(4-Fluorophenyl)-2-(4-methylsulfinylphenyl)-5-(4-pyridyl)1H-  Imidazole |
| SB220025 | 60 mM | 5-(2-Amino-4-pyrimidinyl)-4-(4-fluorophenyl)-1-(4-piperidinlyl)  imidazole |
| PD169316 | 89 nM | 4-(4-Fluorophenyl)-2-(4-nitrophenyl)-5-(4-pyridyl)-1H-imidazole |
| MAPK InhIII | 380 nM | RS)-{4-[5-(Fluorophenyl)-2-methylsulfanyl-3H-imidazol-4-yl]pyridin-2-yl}-(1-phenylethyl)amine] |
