# Supplementary material for: Inhibition of Melanogenesis by the Pyridinyl Imidazole Class of Compounds: Possible Involvement of the Wnt/β-Catenin Signaling Pathway
Source: PLoS One. 2012 Mar 13;7(3):e33021. doi: 10.1371/journal.pone.0033021 (PMC3302780; doi:10.1371/journal.pone.0033021)
Supplement: Table S2 — Sequences real-time PCR oligonucleotide primers list. (DOCX) [file pone.0033021.s004.docx]

**Table S2.** Oligonucleotide primers list.

| **Target gene** | **Forward primer** | **Reverse primer** |
| --- | --- | --- |
| HMitf | 5’-ATGGACGACACCCTTTCTC-3’ | 5’-GGAGGATTCGCTAACAAGTG-3’ |
| HTyr | 5’-GGCCAGCTTTCAGGCAGAGGT-3’ | 5’-TGGTGCTTCATGGGCAAAATC-3’ |
| hTRP1 | 5’-AAGCAGACATCCAACAACACTAG-3’ | 5’-GCAAGAGTTCAGAACACAGGTC-3’ |
| hTRP2 | 5’-GCAAGAGATACACGGAGGAAG-3’ | 5’-CTAAGGCATCATCATCATCACTAC-3’ |
| hActin | 5’-GACAGGATGCAGAAGGAGATTACT-3’ | 5’- TGATCCACATCTGCTGGAAGGT-3’ |
